# Transgender and Nontrans Patients Do Not Receive Statistically Different Quality Primary Care at Whitman-Walker Health, 2008–2016

**DOI:** 10.1089/trgh.2019.0022

**Published:** 2019-09-23

**Authors:** Deborah A. Goldstein, Eleanor Sarkodie, W. David Hardy

**Affiliations:** ^1^ Whitman-Walker Institute, Washington, District of Columbia.; ^2^ Division of Infectious Diseases, Department of Medicine, Georgetown University Medical Center, Washington, District of Columbia.; ^3^ Division of Infectious Diseases, Johns Hopkins University School of Medicine, Baltimore, Maryland.

**Keywords:** HIV, primary care, primary care quality indicator, transgender

## Abstract

**Purpose:** Washington, DC, has the highest prevalence of transgender persons in the United States at 2.8%. Transgender persons in DC have lower income, less stable housing, and more HIV infection than nontrans persons. Data are scarce regarding primary care quality among trans persons. We provide a detailed analysis of transgender patients at Whitman-Walker Health, an HIV- and LGBT-focused community health center.

**Methods:** We performed a retrospective electronic medical record review of transgender patients ≥18 years of age from 2008 to 2016, evaluating demographic factors, HIV status, gender-affirming care, and primary care quality indicators.

**Results:** Of 20,097 patients, 1822 (9.0%) self-identify as transgender (62.9% trans female and 37.2% trans male), and 18,275 were nontransgender. Transgender patients are more likely to be young, white, HIV negative, and reside outside Washington, DC, than nontrans patients. Transgender patients are more likely to engage in primary care and have a similar likelihood of mammogram and colonoscopy screening than nontrans patients. Trans males are more likely to be privately insured, have lower rates of HIV testing than nontrans patients, and have higher rates of cervical Pap smears than cis females. Trans females have a high prevalence of HIV infection (26.6%).

**Conclusion:** This is the largest single-center U.S. transgender cohort to date. Over a quarter of trans females are HIV positive, consistent with a national prevalence of 27.7%. Transgender and nontrans patients do not receive statistically different quality of primary care. Trans patients' high engagement in primary care may result from providing hormone therapy and primary care within a single provider visit.

## Introduction

Washington, DC, has the highest prevalence of transgender persons of any U.S. state at 2.8%^1^; this is also greater than the estimated 0.3–0.5% worldwide prevalence.^2^ Self-administered surveys of DC's trans community reveal less education, lower income, and less stable housing coupled with greater HIV infection, compared to DC's nontrans population.^3^ However, data are lacking on primary care engagement and quality of care among transgender patients.^4^

Transgender persons face significant disparities in access to primary health care, due, in part, to lack of appropriate and competent medical care.^3^ A recent analysis of peer-reviewed transgender health literature from 2008 through 2014 found most studies addressed mental health, sexual health, or substance abuse, while barely 7% of articles focused on general health, including mortality, hormone use, and cancer; researchers concluded that “the general health of transgender people is the least researched aspect of the transgender global burden of disease.”^2^ A second review found no study addressing mammography, colorectal screenings, or influenza vaccinations, and suggested “an urgent need for research that addresses the primary care needs of all transgender and gender-nonconforming people.”^4^

Our motivation is to provide high-quality primary care to transgender patients. Because primary health care of transgender patients is an understudied area with few evidence-based guidelines, we analyzed primary care indicators from a large transgender cohort at Whitman-Walker Health (WWH), an HIV- and LGBT-focused Federally Qualified Health Center in Washington, DC. WWH has offered medical and support services for the trans community since 2005. In 2016, WWH served 8990 unique medical clients, including 1229 transgender (13.6%) and 3285 (37%) HIV-positive patients.

## Methods

We conducted a retrospective electronic medical record (EMR) review between January 1, 2008 and January 1, 2017. We included patients ≥18 years of age who self-identified as transgender on their initial clinic registration form, and who had attended at least one medical visit during the study period at either of WWH's two clinical sites in Washington, DC. WWH's research protocol for a retrospective trans health study was approved by Chespeake IRB in April 2016. This proposal was subsequently modified in November 2016 to broaden study population, dates of analysis, and include primary care quality indicators; this revised proposal was exempted from IRB oversight in December 2016.

WWH's clinic registration form queries transgender identity, but does not elicit specific information about natal sex and current gender identity; transgender identity is not stratified by trans female or trans male. For purposes of analysis, we defined trans female as someone assigned male sex at birth, whose current gender identity is female; trans male as someone assigned female sex at birth, whose current gender identity is male^2^; and nonbinary person as someone who does not identify as trans female or trans male. WWH's EMR documents self-identified transgender patients with legal sex (sex on drivers license or insurance card) followed by “(T).” However, this practice may list gender identity or natal sex instead of legal sex. To create our transgender cohort, we identified all patients with “(T)” in their chart and sorted by hormone use. Those with “(T)” who were prescribed estrogens and/or antiandrogens were considered trans female; those with “(T)” who were prescribed testosterones were considered trans male. We performed chart review for patients with “(T),” who were not prescribed hormone therapy and, based on providers' narrative charting, grouped these patients as trans female, trans male, or nonbinary. Nonbinary patients (*n*=10) were excluded from the analysis.

We evaluated demographic factors, HIV infection, vaccinations, laboratory values, medical visits, and cancer screening among trans and nontrans cohorts, as well as gender-affirming hormone prescriptions and surgical referrals. Self-reported gender identity, race, ethnicity, and housing status from initial clinic registration forms were entered into eClinicalWorks (eCW), and then abstracted using BridgeIT software; SPSS was used for analysis. Age, insurance status at first appointment, residency at last appointment, and appointment data were handled similarly. Primary care indicators of interest were as follows: (1) at least one influenza vaccine during study period; (2) tobacco screening at first medical visit; (3) documentation of current smoking status; (4) at least one lipid screening during study period; (5) referral for mammogram; (6) referral for colonoscopy; and (7) cervical PAP screening. Primary care indicators were abstracted using ICD9, ICD10, and CPT codes, laboratory results, and external referrals.^5^ HIV status was determined by ICD9 and ICD10 codes for HIV infection; HIV status was reported as “unknown” if there were no ICD9 or ICD10 codes for HIV infection and no HIV testing reported in eCW. eCW was queried for medication prescriptions for hormones and gender-affirming surgical referrals, with a focus on medical indication and insurance type. We analyzed mean number of medical appointments during the study period as well as primary care engagement, defined as three or more appointments during the study period.

There are no transgender-specific primary care guidelines. The World Professional Association for Transgender Health^6^ recommends using country-specific guidelines where possible. We utilized recommendations from the University of California, San Francisco Center of Excellence for Transgender Health's Guidelines for the Primary and Gender-Affirming Care of Transgender and Gender Nonbinary People for tobacco screening, colonoscopy, and mammogram recommendations.^7^ We referenced U.S. Preventive Services Task Force (USPSTF) guidelines and WWH's internal protocol for transgender health care for lipid screening.^8^ We utilized USPSTF guidelines for cervical PAP screening^9^ and the 2016 Advisory Committee on Immunization Practices guidelines for influenza vaccination recommendations.^10^

### Statistical analyses

Chi-square analysis was performed between gender identity and most primary care indicators. To better understand the effects of sociodemographic variables on primary care outcomes, the relationship between race, ethnicity, residency at last appointment, and insurance at first appointment and primary care indicators within each gender identity was examined using chi-square. *Post hoc* testing using adjusted standardized residuals was also performed. An analysis of variance (ANOVA) was performed between gender identity and mean medical appointments. An alpha level of 0.05 was used for all statistical tests. Outcomes were compared to national Healthy People 2020 primary care benchmarks.^11^

## Results

### Demographics

**FIG. 1.**
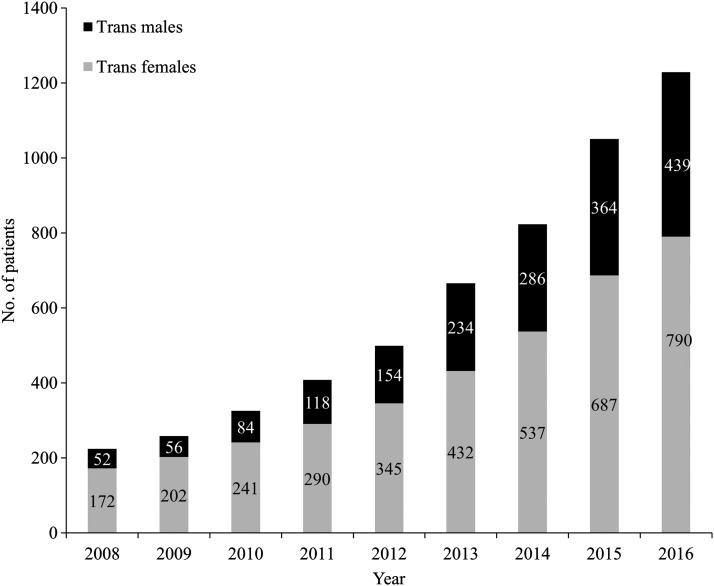
Transgender patients in medical care at WWH by year, 2008–2016. “In medical care” is defined as at least one medical appointment with a WWH provider in a specific calendar year. WWH, Whitman-Walker Health.

Of 20,097 patients seen during 2008–2016, 1822 were transgender (9.0%), with 677 (37.2%) trans males and 1145 (62.9%) trans females; 18,275 were nontransgender (Table 1). Annually, the number of transgender patients receiving care at WWH, defined as at least one medical visit in a calendar year, increased each year during the study period, with 790 trans females and 439 trans males in care in 2016 (Fig. 1). Among trans females, 31.3% identified as African American; 50.8% were between age 25–40; 20.2% identified as Hispanic or Latina; 20.1% reported unstable or temporary housing; and 28.1% had public insurance.

**Table 1.** Demographics of Transgender and Nontransgender Patients at Whitman-Walker Health, January 2008 to January 2017

Total	Transgender females		Transgender males		Nontrans patients		*p*^a^
	*n*	%	*n*	%	*n*	%	
	1145	100.0	677	100.0	18275	100.0	
Age at first appointment							<0.001
18–24	375	32.8	332	49.0	2486	13.6	
25–40	582	50.8	291	43.0	8802	48.2	
41–65	187	16.3	53	7.8	6614	36.2	
>65	1	0.1	1	0.1	373	2.0	
Race							<0.001
White	532	46.5	424	62.6	7070	38.7	
Black/African American	356	31.1	137	20.2	7644	41.8	
Asian	38	3.3	19	2.8	573	3.1	
Other	46	4.0	29	4.3	355	1.9	
Unreported	173	15.1	68	10.0	2633	14.4	
Ethnicity							<0.001
Hispanic or Latino	256	22.4	49	7.2	3071	16.8	
Not Hispanic or Latino	781	68.2	551	81.4	13465	73.7	
Unreported	108	9.4	77	11.4	1739	9.5	
Housing status at first appointment							<0.001
Stable/permanent	777	67.9	515	76.1	11484	62.8	
Unstable/temporary	230	20.1	99	14.6	2436	13.3	
Unreported	138	12.1	63	9.3	4355	23.8	
Insurance at first appointment^b^							<0.001
Private	432	38.6	420	63.2	6419	37.4	
Public	314	28.1	60	9.0	6693	39.0	
Self-pay	231	20.6	128	19.2	2406	14.0	
Other	142	12.7	57	8.6	1636	9.5	
Residency at last appointment, state							—
District of Columbia	547	47.8	205	30.3	13790	75.5	
Maryland	247	21.6	217	32.1	2275	12.4	
Virginia	314	27.4	223	32.9	1906	10.4	
Other Southern states^c^	18	1.6	19	2.8	102	0.6	
Northeast^d^	9	0.8	7	1.0	76	0.4	
Other states^e^	10	0.9	6	0.9	88	0.5	
No data available	0	0.0	0	0.0	38	0.2	
HIV status							<0.001
Positive^f^	305	26.6	7	1.0	6494	35.5	
Negative HIV test	519	45.3	270	39.9	5454	29.8	
Unknown status	321	28.0	400	59.1	6327	34.6	

^a^ A chi-square test of independence at an alpha level of 0.05 was used to obtain all *p*-values reported.

^b^ Reporting insurance status for 97.7% of trans females, 98.2% of trans males, and 93.9% of nontrans patients. Data missing for 1115 participants.

^c^ Other Southern states include the following: DE, *n*=2; WV, *n*=32; KY, *n*=5; TN, *n*=3; NC, *n*=17; SC, *n*=5; GA, *n*=17; FL, *n*=26; AL, *n*=2; MS, *n*=1; LA, *n*=2; OK, *n*=1; and TX, *n*=14 (totals for entire cohort in each state).

^d^ Northeastern states include the following: PA, *n*=32; NJ, *n*=9; CT, *n*=6; MA, *n*=9; NY, *n*=35; and VT, *n*=2 (totals for entire cohort in each state).

^e^ Other states include the following: SD, *n*=1; KS, *n*=1; MN, *n*=1; IA, *n*=2; MO, *n*=6; WI, *n*=3; IL, *n*=11; IN, *n*=1; MI, *n*=3; OH, *n*=3; WA, *n*=8; OR, *n*=2; CA, *n*=28; AK, *n*=1; ID, *n*=1; NV, *n*=3; MT, *n*=1; UT, *n*=2; AZ, *n*=2; CO, *n*=6; NM, *n*=1; and HI, *n*=1; and international countries (totals for entire cohort in each state).

^f^ Number of subjects who were *ever* HIV positive during study period, based on ICD-9 and ICD-10 codes and results of HIV tests in WWHs EMR. Unknown status indicates the absence of HIV testing at WWH during the study period.

EMR, electronic medical record; WWH, Whitman-Walker Health.

Bivariate analysis demonstrated a statistically significant association between age, race, insurance status at first appointment, housing status at first appointment, and gender identity. Trans males are more likely to be between age 18–24 (*p*<0.001); white (*p*<0.001); and privately insured (*p*<0.001); and have stable/permanent housing at their first medical appointment (*p*<0.001) compared to trans females and nontrans patients (Table 1). While most of WWHs nontrans population resided within DC, the majority of trans patients lived outside of Washington, DC (75.5% nontrans, vs. 47.8% trans females and 30.3% trans males), with many traveling from West Virginia, New York, Pennsylvania, North Carolina, Georgia, and Florida (Table 1).

### HIV

Despite high HIV infection rates among trans females, the nontrans population was more likely to be HIV positive when compared to both trans male and trans female patients (*p*<0.001). Trans males were more likely to have unknown HIV status, indicating an absence of HIV testing at WWH during the study period (59.1% of trans males vs. 28.0% of trans females and 34.6% of nontrans) (Table 1).

### Gender-affirming care

More than 90% of trans patients received prescriptions for gender-affirming hormone therapy (94.2% trans females and 91.3% trans males) (Table 2). Of trans females who were prescribed estrogen, 95.7% received estrogen with an antiandrogen and 4.3% received estrogen alone. All trans males on hormone therapy were prescribed testosterone. WWH's protocol for gender-affirming hormone therapy was previously reported.^12^

**Table 2.** Hormone Use by Transgender Patients at Whitman-Walker Health, January 2008 to January 2017

Transgender females	*n*=1145	100%
Estrogen and antiandrogen	1014	88.6
Estrogen alone	46	4.0
Antiandrogens alone	18	1.6
No hormone therapy	67	5.8
Transgender males	*n*=677	100%
Testosterone therapy	618	91.3
No hormone therapy	59	8.7

**FIG. 2.**
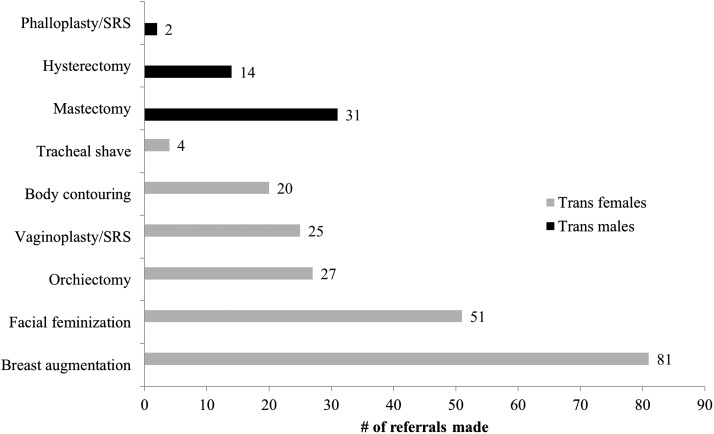
Surgical referrals by indication for gender-affirming care of transgender patients, WWH, 2016.

The proportion of transgender patients receiving surgical referrals for gender-affirming care increased from 2014 to 2016: in 2014, 4.5% received surgical referrals, followed by 11.4% in 2015 and 18.7% in 2016. In 2016, among the 230 trans patients who received surgical referrals for gender-affirming care, 67.8% had Medicaid, 24.3% had private insurance, 5.2% had Medicare, and 2.6% were self-pay. The most common indications for surgical referrals in 2016 were breast augmentation and facial feminization for trans females, and mastectomy and hysterectomy for trans males (Fig. 2).

An ANOVA showed a statistically significant difference in mean medical appointments among the three gender identities [*F*=(2, 20094)=12.93, *p*<0.001; Table 3]. A Tamhane *post hoc* test suggested that the mean number of medical appointments during the study period for nontrans patients (mean=9.64, standard deviation [SD]=12.9) was significantly lower than the mean number of medical appointments for trans female patients (mean=11.38, SD=10.8; *p*<0.001), and mean number of medical appointments for trans males (mean=8.57, SD=7.4) was significantly lower compared with both trans females (*p*<0.001) and nontrans patients (*p*=0.001). Trans female patients had greater engagement in primary care, defined as more than three medical appointments during the study period, than trans male and nontrans patients (*p*<0.001).

**Table 3.** Primary Care Indicators for Transgender Patients at Whitman-Walker Health, January 2008 to January 2017

Total	Transgender females		Transgender males		Nontrans patients			Healthy People 2020 (%)
	*n*	%	*n*	%	*n*	%		
	1145	100.0%	677	100.0%	18275	100.0%	*p*^a^	
Mean, medical appointments	11.4 (SD=10.8)		8.6 (SD=7.4)		9.6 (SD=12.9)		<0.001	
Primary care engagement							<0.001	
>3 Appointments	941	82.2	501	74.0	10209	55.9		
Influenza vaccinations							<0.001	80.0
At least one vaccination	451	39.4	161	23.8	6918	37.9		
Never vaccinated	694	60.6	516	76.2	11357	62.1		
Tobacco screen at baseline							<0.001	68.6
Screened at baseline	999	87.2	604	89.2	12030	65.8		
Never screened	146	12.8	73	10.8	6245	34.2		
Smoking status at baseline^b^							<0.001	
Current smoker	250	25.0	128	21.2	3471	28.9		
Nonsmoker	749	75.0	476	78.8	8559	71.1		
Lipid screenings^c^							<0.001	82.1
At least one screen	826	74.4	539	86.1	11617	64.0		
Never screened	284	25.6	87	13.9	6528	36.0		
Mammogram referrals^d^							0.107	81.1
At least one referral	37	33.9	4	21.1	653	40.2		
Never referred	72	66.1	15	78.9	970	59.8		
Colonoscopy referrals^e^							0.683	70.5
At least one referral	33	30.3	4	21.1	1372	30.3		
Never referred	76	69.7	15	78.9	3159	69.7		
Cervical pap smears^f^							<0.001	93.0
At least one pap smear			337	56.2	3167	49.1		
No pap smear			263	43.8	3278	50.9		

^a^ A chi-square test of independence at an alpha level of 0.05 was used to obtain all *p*-values reported, except for mean medical appointments. For mean medical appointments, the one-way ANOVA test was used to obtain the *p*-value.

^b^ Only includes patients with tobacco screen data.

^c^ Only includes patients who were ever 20 years of age and older during the study period.

^d^ Only includes transgender female, transgender male, and cisgender female patients who were ever 50 years of age and older during the study period.

^e^ Only includes patients who were ever 50 years of age and older during the study period.

^f^ Only includes transgender male and cisgender female patients who were ever 21 years of age and older during the study period.

ANOVA, analysis of variance; SD, standard deviation.

### Primary care indicators

Trans males were less likely to receive at least one influenza vaccine than trans females or nontrans patients (*p*<0.001; Table 3). Trans males were more likely to receive tobacco screening at their first medical visit (*p*<0.001). Nontrans patients were more likely to identify as current smokers (*p*<0.001). Trans male patients were more likely to receive at least one lipid screening than trans females and nontrans patients (*p*<0.001). There was no statistically significant association between gender identity and either mammogram referrals (*p*=0.107), or colonoscopy referrals (*p*=0.683). There is a statistically significant association between cervical pap screening and gender identity, with trans males more likely to have had at least one cervical pap smear than cis females (*p*=0.001). All three groups scored lower than the goals set forth by HealthyPeople2020 for influenza vaccination, mammogram referral, colonoscopy referral, and cervical pap smears^11^ (Table 3).

*Post hoc* analysis of socioeconomic variables among trans females showed: (1) influenza vaccination was greater than expected among African Americans, DC residents, and publicly insured; (2) identification as smokers was greater than expected among African Americans and DC residents; and (3) receipt of at least one lipid screening and referrals for mammogram and colonoscopy were greater than expected among DC residents (Table 4). Among trans males, receipt of at least one cervical Pap smear was greater than expected among DC residents.

**Table 4.** Adjusted Standardized Residuals for Associations Between Sociodemographic Factors and Primary Care Indicators by Gender Identity

	Transgender females						Transgender males^a^	
	Influenza vaccine	Tobacco screen	Smoking status	Lipid screening	Mammograms	Colonoscopy	Influenza vaccine	Cervical pap smears
Race								
White	166 (−5.3)^b^	—^c^	58 (−8.8)	—	—	—	—	209 (0.3)
African American	183 (5.6)	—	147 (11)	—	—	—	—	85 (2.4)
Asian	13 (−0.7)	—	5 (−1)	—	—	—	—	4 (−1.9)
Other	18 (0)	—	6 (−1.7)	—	—	—	—	10 (−2)
Ethnicity								
Hispanic	—	234 (2.3)	—	196 (1.9)	—	—	—	—
Non-Hispanic	—	688 (−2.6)	—	566 (0.3)	—	—	—	—
Residency								
DC resident	271 (6.8)	—	177 (8.2)	426 (4)	24 (2)	23 (2.5)	59 (2)	135 (4.2)
Non-DC resident	180 (−6.8)	—	73 (−8.2)	400 (−4)	13 (−2)	10 (−2.5)	102 (−2)	202 (−4.2)
Insurance								
Private	137 (−4.1)	—	—	289 (−2.7)	—	—	—	—
Public	171 (6.4)	—	—	241 (2.2)	—	—	—	—
Self pay	76 (−2.3)	—	—	164 (−0.5)	—	—	—	—
Other	52 (−0.7)	—	—	110 (1)	—	—	—	—

^a^ Among transgender males, only influenza vaccine and cervical pap smears showed a significant association among primary care indicators.

^b^ Reported results are observed counts with adjusted standardized residuals in parentheses. Adjusted standardized residuals were considered significant if >3 or < −3.

^c^ Observed counts and adjusted standardized residuals were only reported for associations that were statistically significant with a primary care outcome at *p*<0.05.

## Discussion

This is the largest single-center US transgender cohort presented to date, and also the first medical care quality study of mammography, colorectal cancer screening, and influenza vaccination in transgender persons, to the best of our knowledge.^13,14^ This cohort is unique because it is predominantly trans female, privately insured, and has a high proportion of trans females with HIV infection, unlike other well-characterized US transgender cohorts.^13,15^ Compared to WWH's nontrans patients, transgender patients at WWH are more likely to be young, white, privately insured, have stable/permanent housing, and reside outside of Washington, DC. Trans females are more likely to identify as African American and/or Hispanic/Latina and have unstable/temporary housing and public health insurance, while trans males are more likely to identify as white, have stable/permanent housing, and have private health insurance.

### Gender-affirming care

WWH's transgender care model envisions gender-affirming care, including hormone therapy and surgical referrals for gender transition, as an important part of primary care; therefore, WWH's medical providers integrate transgender care into primary care appointments. We hypothesize that transgender patients' high degree of primary care engagement is a reflection of this care model. More than 90% of WWH transgender patients received gender-affirming hormone therapy; similarly, Fenway Health reported 81% of trans males and 89% of trans females received hormonal therapy.^15^ The recent increase in referrals for gender-affirming surgeries is, in large part, due to an updated DC Medicaid policy for coverage of gender reassignment surgery.^16^ The increasing availability of surgical specialists in the Washington, DC, area also influenced this trend.^17^

### HIV testing

The CDC recommends that persons at risk for HIV infection be screened for HIV at least annually, although transgender persons are not specifically discussed in current recommendations.^18^ Some studies have found that transgender patients undergo HIV screening at rates higher than the general population.^4^ However, in a recent survey of transgender respondents, trans females and trans males self-reported HIV testing at levels similar to cisgender heterosexuals, and at levels inconsistent with their HIV risk.^18^ Our data reveal a HIV testing deficit among trans males at WWH. This finding requires additional exploration of the unique testing barriers this population faces, including providers' pre-conceptions about sexual activity and HIV risk.

### HIV infection

The 28% HIV seroprevalence among trans females is consistent with other published U.S. estimates of 10–52.4%.^4^ WWH specializes in providing HIV care, reflected by the large percentage of nontrans HIV+ persons in care. There are little available data regarding HIV seroprevalence among transgender males; the 1.0% HIV seropositivity among trans males in this study is consistent with previous reports of 0–11% HIV positivity.^4^ Many published reports of HIV seroprevalence among transgender persons are based on self-report, which likely underestimates seroprevalence.^4^ Results from the ongoing multi-site LITE study, a longitudinal cohort of HIV-negative trans females in the eastern and southern United States, will help to characterize risk factors for HIV infection and access to HIV prevention methods to inform evidence-based HIV prevention interventions.^19^

### Primary care indicators

Annual influenza vaccination is recommended for all persons ≥6 months of age without contraindications.^10^ Vaccination data among transgender patients are limited. In a recent report of LGBT respondents, 4% of whom identified as transgender, annual influenza vaccination prevalence reached 68.0%.^20^ WWH's low rate of trans male influenza vaccination may be due to privately insured trans males traveling from outside Washington, DC, to receive gender-affirming care at WWH with primary care providers near home who administer influenza vaccinations; these vaccinations are not well captured by our EMR. Alternately, trans males, with low rates of tobacco use and HIV infection, may be perceived as low risk of influenza-related complications and not offered vaccination, or they may refuse vaccination.

Trans female smokers should be counseled on tobacco risks and cessation options at every visit; no specific recommendation is made for trans males.^7^ While previous studies have reported high rates of tobacco use among transgender persons, others comparing smoking rates among transgender people to cisgender lesbian, gay, and bisexual people did not find statistically significant higher rates of smoking.^5^ At WWH, transgender patients were more likely to receive tobacco screening and less likely to identify as current smokers than nontrans patients. This may be because WWH, an LGBT-focused health center, has a relatively high rate of current smokers among nontrans patients, nearing 30%. High lipid screening among trans females and trans males reflects WWH's clinical protocol, which includes baseline lipid panels before hormone initiation for all trans patients and a follow-up lipid panel for trans males 3 months after testosterone initiation. We evaluated lipid screening among those ≥20 years of age during the study period, in keeping with the USPSTFs grade B recommendation.^8^

Clinical guidelines recommend trans females undergo screening mammography every 2 years beginning at the age of 50 and after receiving 5–10 years of feminizing hormones.^7^ Trans males who have not undergone bilateral mastectomy or who have only undergone breast reduction should undergo screening for cis females, although there is scant evidence to support this recommendation.^7^ Fenway Health reported transgender patients, including trans females and trans males without bilateral mastectomy, were less likely than cisgender females to adhere to mammography recommendations.^21^ We restricted our analysis to those ≥50 years of age during the study period; among trans females, we did not account for hormone duration, and among trans males we did not account for history of mastectomy or breast reduction, making it difficult to assess if mammogram referrals were consistent with published guidelines.

Growing literature suggests lower rates of cervical Pap tests among transgender males^2^ due to barriers to screening, including vaginal atrophy and dryness causing discomfort during examination.^22^ Fenway Health-reported trans males were less likely to be up to date on Pap tests than nontrans females.^21^ Our higher trans male Pap rate may reflect provider commitment to screen this population and secure documentation of Paps from outside providers. WWH Pap rates among both trans males and cis females are considerably lower than the HealthyPeople2020 benchmark and represent future areas for improvement.

DC residents' improved primary care outcomes may be due to broad availability of DC Medicaid or to underdocumentation of primary care interventions performed on non-DC-resident patients by health care providers outside of WWH.

### Limitations

This retrospective EMR review is subject to bias from convenience sampling of our clinic population; inadequacy in capturing health data outside of our health system; and limitations of an EMR designed for a cisgender population. Our transgender cohort's predominant composition of young, white, HIV–negative, and privately insured patients suggests access to care not reported in previous transgender cohorts, and this impacts evaluation of primary care outcomes. It is difficult to capture the dynamic process of gender transition in a retrospective chart review. We did not match trans to cis gender patients, but instead compared transgender patients to a large mixed sample of nontrans patients. Our analysis of referrals for cancer screening instead of completion of screening is problematic and reflects the limitations of available EMR data; this methodology does not account for barriers in completing these examinations, including transphobic providers and fear of being misgendered. The underrepresentation of nonbinary patients is due to an insufficient method of identifying nonbinary patients in our EMR. HealthyPeople2020 indicators were developed for cisgender patients; their use as a guideline for transgender patients is problematic.

## Conclusion

To establish comprehensive, evidence-based national guidelines for the primary care of transgender patients, additional research is needed to determine best practices. There is a need for prospective cohort studies to evaluate optimal age of initiation and optimal interval for repeating cancer screening in transgender patients on hormone therapy, including mammogram and cervical Pap smears. EMRs must strive to adequately document gender identity. Prospective interventions to increase HIV testing among trans males are imperative.

## Abbreviations Used

ANOVA: analysis of variance
eCW: eClinicalWorks
EMR: electronic medical record
SD: standard deviation
USPSTF: U.S. Preventive Services Task Force
WWH: Whitman-Walker Health
